# Cervicomediastinal Hematoma: Atypical Presentation of a Parathyroid Carcinoma

**DOI:** 10.1210/jcemcr/luae063

**Published:** 2024-04-18

**Authors:** Martina Cicia, Giampaolo Papi, Alfredo Scillitani, Stefania Corrado, Pietro Locantore, Alfredo Pontecorvi

**Affiliations:** Unit of Endocrinology, Department of Translational Medicine and Surgery, Università Cattolica del Sacro Cuore, Fondazione Policlinico “A. Gemelli” IRCCS, 00168 Rome, Italy; Endocrinology Unit, Azienda USL Modena, 41121 Modena, Italy; Endocrinology Unit, Casa Sollievo della Sofferenza, 71013 San Giovanni Rotondo (FG), Italy; Pathology Unit, University of Modena and Reggio Emilia, 41125 Modena, Italy; Unit of Endocrinology, Department of Translational Medicine and Surgery, Università Cattolica del Sacro Cuore, Fondazione Policlinico “A. Gemelli” IRCCS, 00168 Rome, Italy; Unit of Endocrinology, Department of Translational Medicine and Surgery, Università Cattolica del Sacro Cuore, Fondazione Policlinico “A. Gemelli” IRCCS, 00168 Rome, Italy

**Keywords:** parathyroid carcinoma, Hashimoto thyroiditis, hematoma, neck mass, *CDC73* gene, Capps’ triad

## Abstract

Parathyroid carcinoma (PC) is a rare endocrine neoplasm that typically presents with osteopenia/osteoporosis, nephrolithiasis, asthenia, and neuropsychiatric symptoms. We describe the case of a 48-year-old woman, presenting with a large painful hematoma in the cervicomediastinal area. The neck ultrasound (US) demonstrated a solid lesion measuring 40 × 80 × 55 mm, markedly hypoechoic, which extended from the right thyroid lobe to the mediastinum. The blood tests showed elevated serum calcium and parathyroid hormone (PTH) concentrations, consistent with hypercalcemic primary hyperparathyroidism. The patient was rehydrated and treated with furosemide, cholecalciferol, and bisphosphonate, and underwent right lower parathyroidectomy, right hemithyroidectomy, and lymphadenectomy of the right VI cervical level. Histological examination was diagnostic for nonangioinvasive or neuroinvasive PC, and the thyroid lobe was the site of lymphocytic thyroiditis; all removed lymph nodes were benign. The postoperative course was regular. Postoperative neck US showed a hypoechoic left thyroid lobe without evidence of residual neoplasm in the right thyroid bed. Levothyroxine therapy of 50 mcg/day was started because of serum thyrotropin concentrations elevated at 18 mcIU/mL (normal reference range, 0.35-4.0 mIU/mL). Eight years after diagnosis, the patient is in good general condition, with no clinical, biochemical, or imaging evidence of disease persistence/recurrence.

## Introduction

Parathyroid carcinoma (PC) is a rare endocrine neoplasm accounting for less than 1% of all forms of primary hyperparathyroidism [1]. The annual incidence of PC ranges from 10 to 13 cases per 10 million per year since 2001, presents equally in males and females (unlike parathyroid adenoma, which is more common in females), and the median age of presentation is 44 to 65 years [2, 3]. Compared to parathyroid adenoma, PC is characterized by much higher serum parathyroid hormone (PTH) and calcium levels [4]. From a clinical point of view, PC typically presents with osteopenia/osteoporosis, nephrolithiasis, asthenia, and neuropsychiatric symptoms [5]. Here, we report the unusual presentation of a PC and discuss the related clinical features.

## Case Presentation

**Figure 1.**
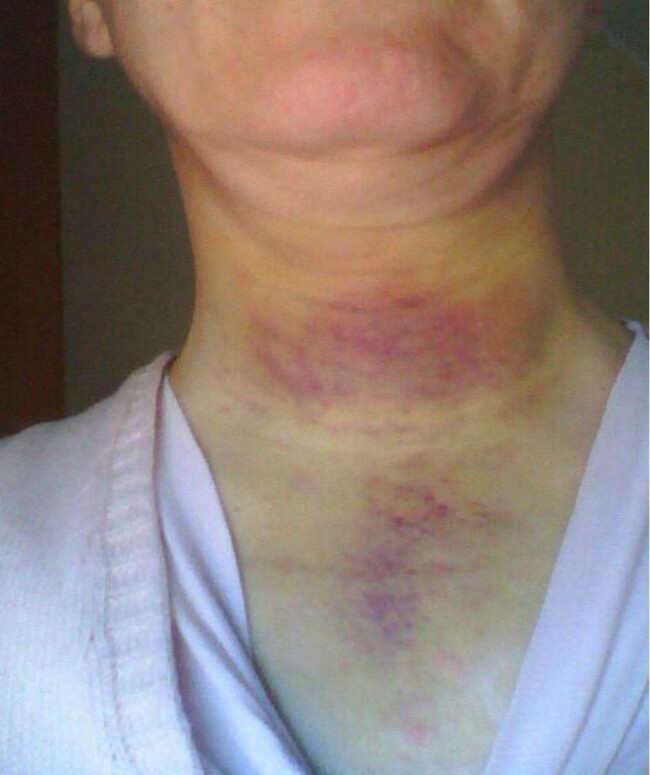
Large hematoma located in the cervicomediastinum associated with swelling in the anterior region of the neck and the mediastinum.

We describe the case of a 48-year-old woman—with no personal or family history of significant diseases—who came to our attention in 2016, the day after the sudden appearance of a large painful hematoma in the cervicomediastinal area (Fig. 1). She did not complain of sore throat, trauma, or difficulty breathing, and was not taking any medication. She reported a 2-month history of a slowly worsening polyuria-polydipsia syndrome.

## Diagnostic Assessment

**Figure 2.**
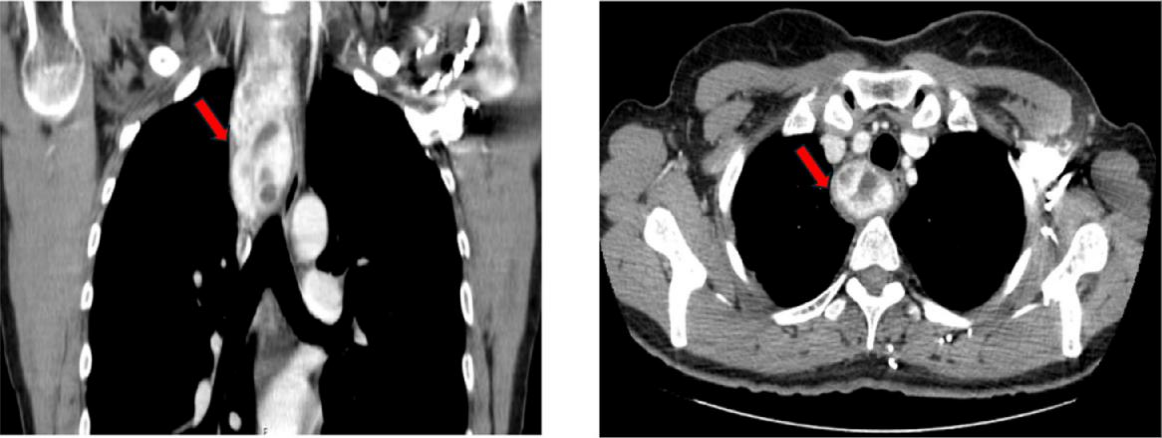
Neck and chest computed tomography. Huge cervicomediastinal mass extending up to the right upper bronchus and contralateral deviation of the trachea and the esophagus.

Ultrasound (US) of the neck showed a huge (40 × 80 × 55 mm) solid, markedly hypoechoic lesion that extended from the right thyroid lobe to the mediastinum. There was a discrete hypoechoic area consistent with blood leakage between the lower pole of the right thyroid lobe and the esophagus. The thyroid gland displayed a diffusely hypoechoic structure, without nodules. The results of laboratory tests are summarized in Table 1. Electrocardiogram did not show significant changes. Lumbar spine and femoral neck dual energy x-ray absorptiometry detected a picture of osteopenia. Renal US showed a 9-mm stone within the left kidney. Computed tomography of either the neck or the mediastinum confirmed the presence of the huge cervicomediastinal mass extending up to the right upper bronchus and displacing the esophagus and the trachea anteriorly and to the left (Fig. 2).

**Table 1.** Results of preoperative laboratory tests

Laboratory test	Results (normal range)
**TSH**	2.1 mIU/mL (0.35-4.0 mUI/mL)
**Calcitonin**	<1 pg/mL (0-7.5 pg/mL)
**PTH**	460 pg/mL (460 ng/L, 1689 pmol/L), (10-65 pg/mL; 10-65 ng/L, 37-239 pmol/L)
**Total serum calcium**	14 mg/dL (3.5 mmol/L) (8.5-10 mg/dL; 2.20-2.60 mmol/L)
**Calcium ion**	2.1 mmol/L (1.17-1.30 mmol/L)
**Calciuria**	535 mg/24 h (29.7 μmol/mol) (60-300 mg/24 h; 5.55-13.9 μmol/mol)
**Serum phosphorus**	2.5 mg/dL (0.81 mmol/L) (2.5-4 mg/dL; 1.0-1.5 mmol/L)
**Serum creatinine**	0.65 mg/dL (49.57 μmol/L) (0.6-1.2 mg/dL; 50-81 μmol/L)
**25-OH-vitamin D**	10 ng/mL (24.96 nmol/L) (30-100 ng/mL; 75-200 nmol/L)
**Carcinoembryonic antigen**	2 ng/mL (0-5 ng/mL)

Abbreviations: PTH, parathyroid hormone; TSH, thyrotropin.

## Treatment

**Figure 3.**
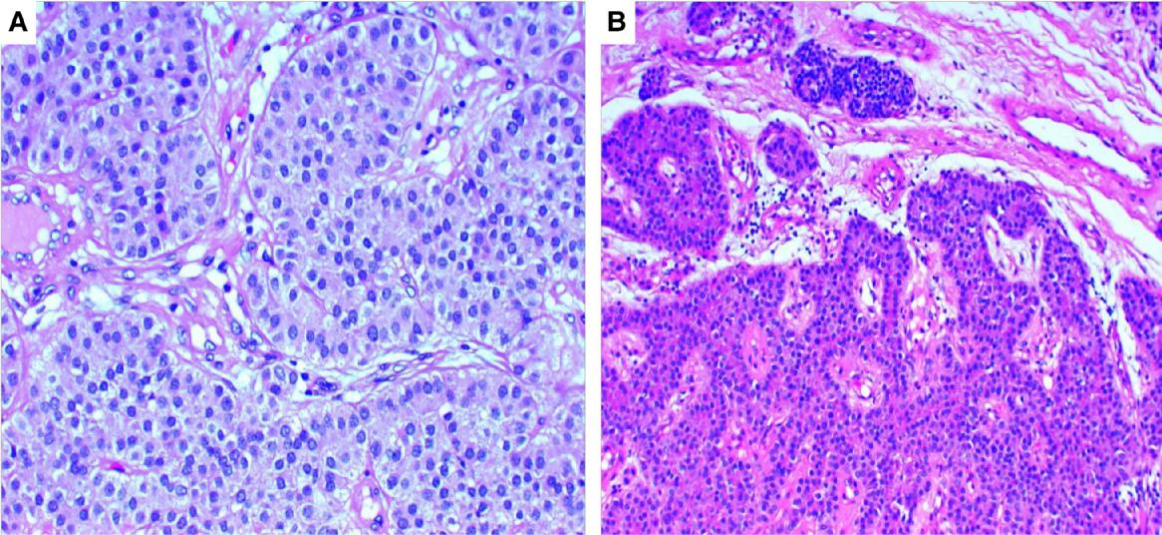
A, Parathyroid neoplasm with areas of glandular structure and trabecular areas without cytological atypia. B, The neoplasm partly showed expansive borders, but in some areas there was evidence of infiltration of the surrounding fibroadipose tissue.

The patient was rehydrated with sodium chloride 0.9% solutions and treated with furosemide 25 mg/day, cholecalciferol 4.000 U/day, and sodium clodronate 100 mg every 10 days/14 days. Because of the high risk of PC, fine-needle aspiration biopsy was not performed and, 4 weeks following symptom appearance, she underwent right lower parathyroidectomy, right hemithyroidectomy, and lymphadenectomy of the right VI cervical level. The histological examination was diagnostic for PC. The neoplasm measured 7 cm on the major axis (macroscopic measurement) and reached the resection margins for and extension of approximately 1.2 cm. The tumor was characterized by areas of glandular structure and trabecular areas without cytological atypia (Fig. 3A). There was evidence of infiltration of the surrounding fibroadipose tissue (Fig. 3B) without vascular or perineural invasion (the latter was also investigated with immunohistochemical reactions for S100 protein and CD31). Isolated shoots and fibrotic areas with focal hemosiderin deposits were observed, too. The right thyroid lobe was the site of lymphocytic thyroiditis; all removed lymph nodes were benign.

## Outcome and Follow-Up

The patient’s postoperative course was regular, with serum calcium and PTH levels of 8 mg/dL (2 mmol/L) (normal reference range, 8.5-10 mg/dL; 2.20-2.60 mmol/L) and 11 pg/mL (11 ng/L) (normal reference range, 10-65 pg/mL; 10-65 ng/L), respectively. The patient was discharged with calcitriol 0.25 mcg/day and calcium carbonate 1000 mg/day therapy. The search for either germline or somatic mutations of the *CDC73* gene was negative. The 30-day follow-up included blood tests and imaging examinations (Table 2). Neck US showed a hypoechoic left thyroid lobe without evidence of residual neoplasm in the right thyroid bed. A 99mTc-MIBI parathyroid scan was negative. Therapy with levothyroxine 50 mcg/day was undertaken, and therapy with calcium and calcitriol was first reduced and then withdrawn after 2 months. Eight years after diagnosis, the patient is in good general condition, with no clinical, biochemical, or imaging evidence of disease persistence/recurrence.

**Table 2.** Results of laboratory tests 30 days after surgery

Laboratory test	Results (normal range)
**TSH**	18 mIU/mL (0.35-4.0 mIU/mL)
**Free thyroxine**	6.2 pg/mL (7.9 pmol/L) (6-12 pg/mL; 9-23 pmol/L)
**PTH**	20.3 pg/mL (20.3 ng/L, 77.4 pmol/L) (10-65 pg/mL; 10-65 ng/L, 37-239 pmol/L)
**Total serum calcium**	8.8 mg/dL (3.49 mmol/L) (8.5-10 mg/dL; 2.20-2.60 mmol/L)
**Serum phosphorus**	2.8 mg/dL (0.9 mmol/L) (2.5-4 mg/dL; 1.0-1.5 mmol/L)
**Alkaline phosphatase**	67 IU/L (9.38 U/dL) (50-220 IU/L; 5-20 U/dL)
**Plasma glucose**	88 mg/dL (4.88 mmol/L) (70-100 mg/dL; 3.3-5.5 mmol/L)
**Antithyroglobulin antibodies**	150 IU/mL (<116 IU/mL)
**Antithyroperoxidase antibodies**	360 IU/mL (<35 IU/mL)

Abbreviations: PTH, parathyroid hormone; TSH, thyrotropin.

## Discussion

We have described the unique case of a PC presenting with pain and hematoma in either the anterior neck and the mediastinum, associated with autoimmune thyroiditis.

In 1932, Capps [6] published the case of a patient with PC manifesting as a cervical-mediastinal painful hematoma and causing the compression and deviation of the esophagus and the trachea. Since then, the association of hematoma involving the neck and the mediastinum, compression of the esophagus and trachea, and the anterior deviation of the trachea by a mass is named “the Capps’ triad.” Patients may complain of neck swelling, shortness of breath, or sore throat. Yet, such a presentation is unusual in patients affected by PC, who most commonly manifest signs and symptoms of bone involvement—such as osteoporosis/osteopenia, fractures, and bone plus muscle pain/weakness (45.8%), or renal damage—including kidney/urinary stones and renal failure (37.2%), fatigue (13.6%) or neuropsychiatric symptoms (11.2%) [5, 7]. Actually, PC is an entity of primary hyperparathyroidism.

A neck mass is palpable in only 11.9% of PC cases. Differential diagnosis includes thyroid and parathyroid nodule, thyroglossal duct lesion, lymphoma, sarcoma, paraganglioma, brachial cyst, derma cyst, sebaceous cyst, metastasis for cervical of noncervical neoplasm, and lymph node swelling due to metastatic or infectious disease (HIV, mononucleosis, tuberculosis, etc) [8]. Any of these entities may provoke a cervical hematoma. However, the main causes of spontaneous cervical bleeding are the following: aneurysm rupture, anticoagulant use, retropharyngeal abscess, mediastinitis, laryngocele, and thyroid or parathyroid nodule bleeding [9]. In particular, a number of cases of cervical hematoma by parathyroid adenoma bleeding have been reported in the literature [10, 11]. In our patient, the suspicion of the parathyroid origin of the mass resulted from elevated serum calcium (>12 mg/dL; > 2.99 mmol/L) and PTH concentrations (>400 pg/mL; > 400 ng/L).

Importantly, we did not perform fine-needle aspiration biopsy in the diagnostic workup due to fears of disrupting the tumor capsule and increasing the possibility of tumor implantation (parathyromatosis) [12]. Indeed, the capsule of parathyroid glands—regardless of their benign or malignant origin—is typically thin and this characteristic may represent the main cause of bleeding in some PC cases, our patient's one included. In particular, the etiological mechanism underlying this event may be attributable to insufficient vascularization of the nodule due to its rapid growth, consequent internal hemorrhage, and capsular rupture [13].

It has been demonstrated that the ideal timing for surgery for parathyroid-related hematoma is 3 months from the acute insult [13]. Indeed, early intervention may be complicated by injury to the recurrent laryngeal nerve or incomplete resection of the affected parathyroid gland. On the contrary, delayed parathyroidectomy leads to improved outcome [13]. Because of the high suspicion of PC, our patient underwent surgery 4 weeks after the onset of symptoms, without complications.

The histological picture of our patient's PC lacked features of aggressiveness, and the search for either germline and somatic mutations of the *CDC73* gene was negative. The latter is a tumor suppressor gene encoding parafibromin, recognized as playing an important role in molecular pathogenesis and associated with the predisposition to certain sporadic PC, as well as hereditary syndromes such as hyperparathyroidism-jaw tumor syndrome (HPT-JT) [14]. Accordingly, laboratory tests ruled out the association with pituitary or pancreatic tumors peculiar to multiple endocrine neoplasia (MEN).

Finally, in our patient US and laboratory tests were consistent with concurrent chronic autoimmune (Hashimoto) thyroiditis (HT). Owing to the high prevalence of HT in the adult population [15], we believe that the association of PC and HT in this case does not imply an etiopathogenetic link, and should be regarded as a casual event.

In conclusion, we have described the unique case of a PC presenting with pain and hematoma both in the anterior neck and the mediastinum, associated with HT. Although parathyroid hemorrhage is a rare event, it should always be suspected when a painful swelling suddenly appears in the neck region in a patient with hypercalcemia.

## Learning Points

- Neck hematoma may be the first clinical manifestation of PC.
- The thin capsule peculiar to parathyroid glands may represent the main cause of bleeding of parathyroid neoplasms.
- The complete surgical removal of PC is crucial for favorable outcome.
- PC can be associated with autoimmune thyroiditis; however, such an association should be regarded as a casual event.

## Contributors

All authors made individual contributions to this manuscript. G.P. and M.C. were involved in the diagnosis and the management of the patient, and in the submission of the manuscript. P.L. and A.P. were involved in the scientific revision of the paper. S.C. performed histological diagnosis and provided the related pictures. A.S. performed genetic examinations (mainly, the search for germline mutation of the *CDC73* gene). All authors reviewed and approved the final draft.

## Data Availability

Original data generated and analyzed during this study are included in this published article.

## Abbreviations

HT: Hashimoto thyroiditis
PC: parathyroid carcinoma
PTH: parathyroid hormone
TSH: thyrotropin
US: ultrasound
